# Liver transplantation in hepatocellular carcinoma – should we perform downstaging?

**DOI:** 10.3325/cmj.2022.63.317

**Published:** 2022-08

**Authors:** Tajana Filipec Kanižaj, Petra Dinjar Kujundžić, Ana Ostojić, Maja Mijić, Helga Sertić Milić, Ana Mijić, Matija Mateljak, Dora Martinčević, Eva Radetić, Vinko Vidjak, Branislav Kocman, Ivana Mikolašević

**Affiliations:** 1 Department of Gastroenterology, Merkur University Hospital, University of Zagreb, School of Medicine, Zagreb, Croatia; 2 Department of Gastroenterology, Merkur University Hospital, Zagreb, Croatia; 3 Department of Gastroenterology, University Hospital Center Zagreb, University of Zagreb, School of Medicine, Zagreb, Croatia; 4 Clinical Department of Diagnostic and Interventional Radiology, Merkur University Hospital, Zagreb, Croatia; 5 Department of Radiology, University Hospital Center Zagreb, Zagreb, Croatia; 6 University of Zagreb, School of Medicine, Zagreb, Croatia; 7 Department of Ophthalmology, Sveti Duh University Hospital, Zagreb, Croatia; 8 Clinical Department of Diagnostic and Interventional Radiology, Merkur University Hospital, University of Zagreb, School of Medicine, Zagreb, Croatia; 9 Department of Surgery, Transplant Center, Merkur University Hospital, Zagreb, Croatia; 10 Department of Gastroenterology, Clinical Hospital Centre Rijeka, School of Medicine, University of Rijeka, Rijeka, Croatia

## Abstract

**Aim:**

To compare the long-term outcomes between liver transplant (LT) recipients with hepatocellular carcinoma (HCC) who were downstaged with transarterial-chemoembolization (TACE) to the Milan criteria (MC) and those initially meeting the MC.

**Methods:**

This retrospective study enrolled 198 patients with HCC: 38 were downstaged and 160 patients initially met the MC. Post-LT survival and HCC recurrence-free survival were evaluated. We assessed the association of death and HCC recurrence with TACE, baseline (age, sex, disease etiology, Model of End-stage Liver Disease, tumor number and the sum of maximum tumor diameters, waiting time, alpha-fetoprotein level) and explant characteristics (tumor number and the sum of maximum tumor diameters, micro- and macrovascular invasion).

**Results:**

The recipient survival rates one, three, and five years after LT were 88.2%, 80.1%, and 75.9%, respectively. HCC recurrence-free probabilities were 92.3%, 87.9%, and 85%, respectively. The outcomes were comparable between the groups. In multivariate analysis, the number of tumors on the explant, age, and tumor recurrence were independent risk factors for death. Only the sum of maximum tumor diameters on the explant was an independent risk factor for HCC recurrence.

**Conclusions:**

Patients successfully downstaged with TACE to the MC can achieve post-LT recipient and HCC recurrence-free survival comparable with patients initially within the MC. Good response to TACE as a criterion for LT may be a method of selecting patients with favorable biological characteristics.

Patients with hepatocellular carcinoma (HCC) and liver cirrhosis constitute 30%-44% of all liver-transplant (LT) candidates in European countries (1). In most western LT programs, high cure rates of HCC are a direct consequence of strict pretransplant selection criteria, combination of known tumor size and number, as well as the application of the validated Milan criteria (MC) (2). Still, around 70% of HCC patients are diagnosed with extensive disease, which makes them unsuitable for this curative intervention (3). In order to increase the pool of recipients with an acceptable post-LT outcome, some centers use more advanced selection criteria, while others perform tumor downstaging with loco-regional therapies (LRT) (1,4-6). In most studies, tumor reduction to the fulfillment of the MC based on radiographic findings is considered as successful downstaging. Data about the effects of downstaging on the outcome of LT are discrepant and mostly provided by uncontrolled studies (7). There is no common or even majority agreement regarding the optimal LRT method, patient selection criteria, treatment end-points, response assessment protocols, or a minimum observation period from downstaging to LT (1,4,8). The aim of this study was to compare the long-term survival and risk of tumor recurrence between transplanted HCC patients initially meeting the MC and those transplanted after downstaging with transarterial-chemoembolization (TACE).

## Patients and methods

This retrospective, single-center cohort study enrolled 198 adults with HCC and cirrhosis who underwent LT in Merkur University Hospital (MUH), Zagreb, between January 2006 and September 2018. Patients' data were extracted from a prospectively collected database comprising information about all 1152 patients transplanted in MUH during the research period. The inclusion criterion was HCC as an indication for LT in the observed period. Overall, 198/1152 (17.2%) LT recipients met this criterion. Most of them (160/198, 80.8%) initially fulfilled the MC (MC group), while others (38/198, 19.2%) were downstaged to MC before eligibility assessment for LT (downstaging group). The post-LT outcome of the downstaging group was compared with that of patients initially meeting the MC.

The research was approved by the Ethics Committee of MUH and it conformed to the International Conference on Harmonization guidelines on Good Clinical Practice and to the Declaration of Helsinki.

HCC was diagnosed according to the European Association for the Study of the Liver (EASL) guidelines (1). LT exclusion criteria were evidence of extrahepatic malignant disease, macrovascular invasion, or any other standard contraindications against LT (9). Tumor burden was estimated with multiphasic double-contrast spiral computed tomography (CT) and/or magnetic resonance (MR) scans according to standard protocols (Primovist and Xenetix contrast media). Viable tumor number and maximum tumor diameters (MTD) before LT were summarized as the total number of tumors (NT) and the sum of MTD. When the results of two imaging methods were available, the higher number of tumors and the higher sum of MTD was included in the analysis. Downstaging was performed according to the standard TACE protocol. In >90% of patients, drug-eluting beads (DEB) TACE was the procedure of choice (10,11). The eligibility criterion for downstaging was a tumor extending the MC at diagnosis. We defined no upper limits of tumor dimensions or number, time between or the number of iterations of TACE sessions, and the minimum observation period between successful downstaging and listing. Response to LRT was evaluated according to the modified Response Evaluation Criteria in Solid Tumors 1 month after TACE and at a minimum of every 3 months (12).

Pre-transplant data included age, sex, waiting time for LT, Model of End-Stage Liver Disease (MELD) laboratory finding, baseline etiology of cirrhosis, and last recorded alpha-fetoprotein (AFP) level. According to the pretransplant AFP level, patients were divided to six groups (<7, 7-50, 51-100, 101-400, 401-1000, >1000 μg/L) and according to the etiology of cirrhosis to five groups (cryptogenic and non-alcoholic steatohepatitis [NASH], hepatitis B, hepatitis C [HCV], alcoholic, other). The following explant histopathologic characteristics of HCC were recorded: the sum of maximum viable tumor diameters (MTD-3), number of viable tumor nodules (NT-3), MC fulfillment on the explant (Milan-3), and macrovascular (MaVI) and microvascular (MiVI) invasion.

### Statistical analysis

Baseline characteristics are presented as median and interquartile range (IQR). Quantitative variables were compared with the *t* test or Mann-Whitney test, whereas qualitative variables were compared with the χ^2^ or exact tests (Fisher exact test or likelihood ratio test). The associations of the tested variables with survival and tumor recurrence risk were evaluated with multivariate Cox regression analysis (backward stepwise method). The receiver operating curve (ROC) curve analysis was used to establish the optimal cut-off values of different tumor-related variables for the prediction of tumor recurrence. The influence of various variables on overall survival and recurrence-free survival was evaluated by the Kaplan-Meier method combined with the log-rank test. For survival calculations, LT time was the starting point. *P* < 0.05 was considered significant. Statistical analysis was performed with the Medcalc program (MedCalc Software Ltd, Ostend, Belgium).

## Results

### Baseline characteristics

The study enrolled 198 patients. The median age was 61 years (IQR 57-65); 80.3% patients were men. The most common etiology of liver cirrhosis was alcoholic liver disease (45.5%), followed by HCV (27.8%) and cryptogenic/NASH disease (10.1%). The median value of laboratory MELD at LT was 12 (IQR 9-16). The waiting time to LT was short (median 22.5 days, IQR 7-42).

Downstaging was performed in 38 (19.2%) of patients. Eleven of them were transplanted in <3 months after downstaging. Most (21 patients) underwent only one procedure, 8 underwent three, 7 two, 1 four, and 1 underwent five procedures. Based on the finding of the last pretransplant imaging methods, all patients were within the MC at registration to LT list.

The overall median post-LT follow-up was 1115 days (IQR 506-1904), during which 11.1% patients developed tumor recurrence and 18.6% died. According to the explants finding, 36.9% patients had MiVI, 8.6% MaVI, while 36.9% were outside the MC. The downstaging group had more tumors and a greater sum of MTD at the beginning of downstaging treatment (Table 1).

**Table 1:** Baseline characteristics of the downstaging and Milan criteria groups

	Downstaging (n = 38)	Milan criteria (n = 160)	p
Age (years), median (IQR)	62 (58-65)	61 (56-65)	0.2908
Sex, n (%)			0.8263
male	31 (81.6)	128 (80)	
female	7 (18.4)	32 (20)	
Etiology of cirrhosis, n (%)			0.2960
cryptogenic and non-alcoholic steatohepatitis	6 (15.8)	14 (8.7)	
hepatitis B virus	2 (5.3)	17 (10.6)	
hepatitis C virus	7 (18.4)	48 (30)	
alcohol	19 (50)	71 (44.4)	
other	4 (10.5)	10 (6.3)	
Laboratory MELD, median (IQR)	9.5 (8-15)	12 (9-16)	0.3324
AFP (μg/L), n (%)			0.0861
<7	11 (28.9)	69 (43.1)	
7-50	15 (39.5)	38 (23.8)	
51-100	2 (5.3)	12 (7.5)	
101-400	2 (5.3)	14 (8.8)	
401-1000	2 (5.3)	6 (3.8)	
>1000	6 (15.7)	9 (5.6)	
no data	0	12 (7.5)	
NT, n (%)			0.0054
1	19 (50)	104 (65)	
2	9 (23.7)	26 (16.3)	
3	7 (18.4)	30 (18.7)	
4	0	0	
5	1 (2.6)	0	
6	2 (5.3)	0	
MTD (mm), median (IQR)	64 (46.5-80)	35 (21-48)	<0.0001
Time on waiting list (days), median, (IQR)	19 (9-33)	24 (7-44)	0.4082
NT-3, n (%)			0.0122
1	10 (26.4)	78 (48.8)	
2	7 (18.4)	33 (20.6)	
3	9 (23.7)	19 (11.9)	
>3	12 (31.5)	30 (18.7)	
MTD-3 (mm), median (IQR)	54 (24.5-85)	40 (25-65)	0.1895
Milan-3 no, n (%)	20 (52.6)	53 (33.1)	0.0254
MiVI yes, n (%)	15 (39.5)	58 (36.2)	0.7119
MaVI yes, n (%)	2 (5.3)	15 (9.4)	0.4172

*Abbreviations: IQR – interquartile range, MELD – Model of End-stage Liver Disease; NT – number of tumors; MTD – sum of maximum tumor diameters; MiVI – microvascular invasion; MaVI – macrovascular invasion; NT-3 – number of tumors according to explant histopathological finding; MTD-3 – sum of maximum viable tumor diameters according to explant histopathological finding; Milan-3 – patients not fulfilling the Milan criteria according to explant histopathological finding.

### Post-transplant survival and HCC recurrence

**Figure 1:**
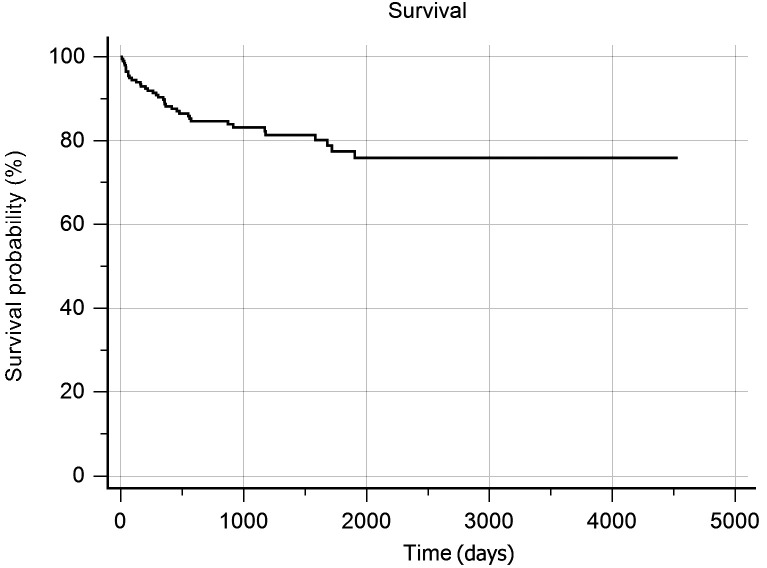
Kaplan-Meier analysis of recipient survival.

**Figure 2:**
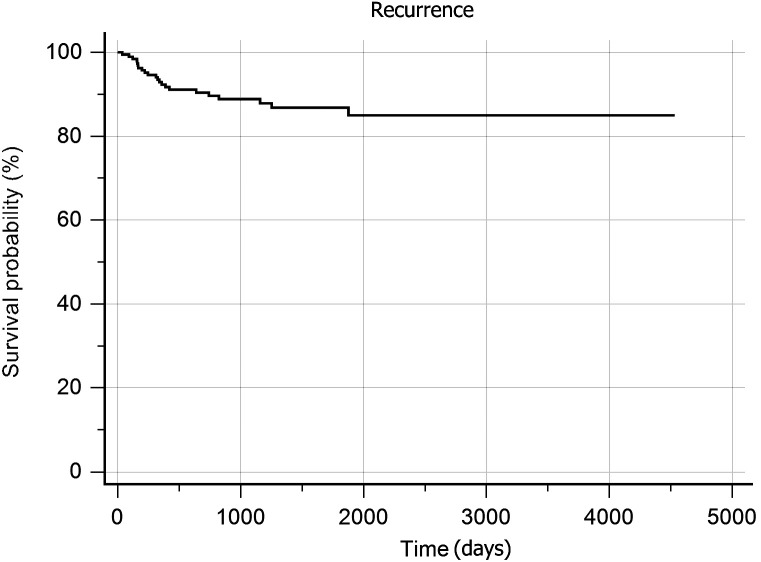
Kaplan-Meier analysis of hepatocellular carcinoma recurrence-free survival.

One, three, and five years after LT, the recipient survival rates were 88.2%, 80.1%, and 75.9%, respectively (Figure 1), and HCC recurrence-free rates were 92.3%, 87.9%, and 85%, respectively (Figure 2).

**Figure 3:**
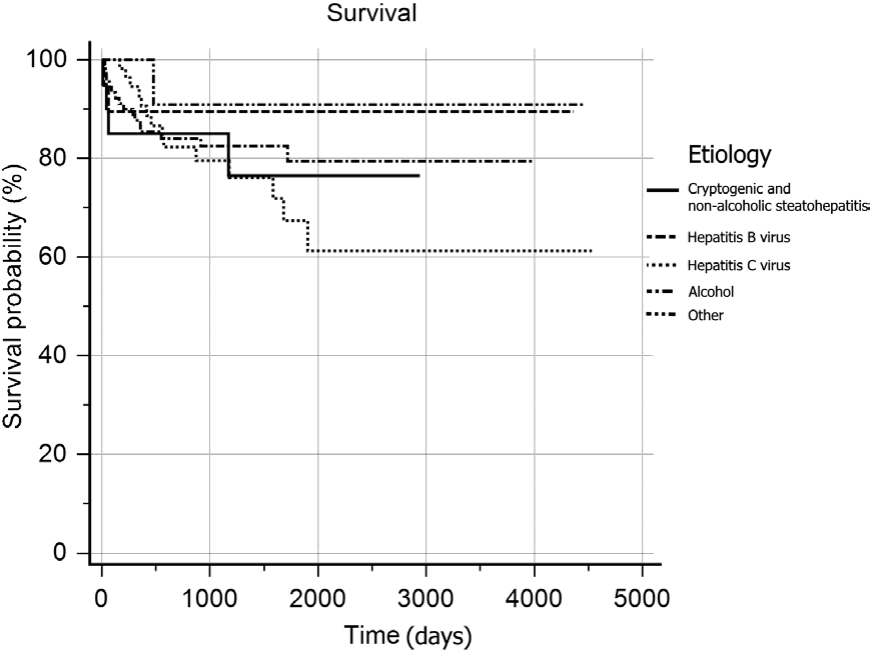
Trend toward lower survival rates of patients with hepatitis C virus and cryptogenic/non-alcoholic steatohepatitis (NASH) cirrhosis, *P* = 0.5576.

Overall, 38% of deaths occurred in the first 6 months, and 60% in the first year after LT. Only 32.4% of all deaths were related to HCC recurrence; 27% of deaths in the first year and 40% later on (*P* = 0.4232). Downstaging did not affect the time and cause of death. Laboratory MELD did not differ between survivors and non-survivors (median [IQR], 12 [9-16] vs 12 [8-16], *P* = 0.8646). Although the difference was not significant, HCV infection and cryptogenic/NASH patients experienced death outcome more frequently than patients with other etiologies (*P* = 0.5576) (Figure 3).

**Figure 4:**
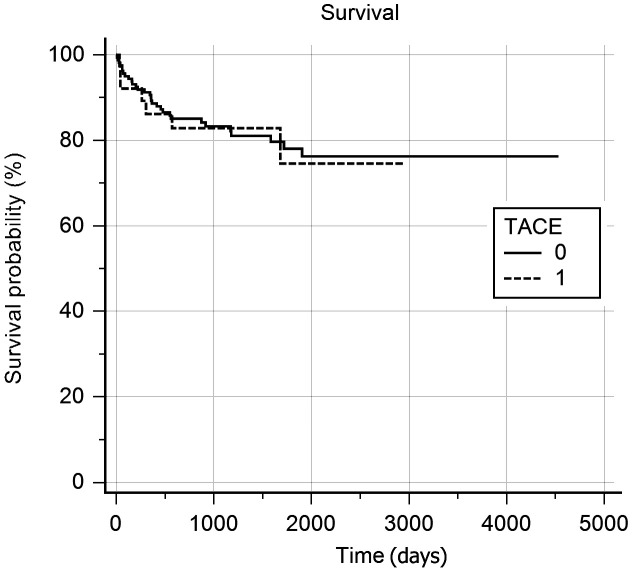
Kaplan-Meier analysis of recipient survival according to downstaging (transarterial chemoembolization) procedure (0 – no, 1 – yes), *P* = 0.8312.

The median post-LT follow-up in the downstaging group was 997 days (IQR 305-1681), during which 18.4% patients developed tumor recurrence and 18.4% died. In the MC group, the median post-LT follow-up was 1115 days (IQR 523-2012), during which 9.4% patients developed tumor recurrence and 18.8% died. Recipient one-year, three-year, and five-year post-LT survival rates did not significantly differ between the groups (downstaging: 88.6%, 81.1%, 76.3% vs MC: 86.2% 81.3%, 75.9%; *P* = 0.8312, Figure 4).

**Figure 5:**
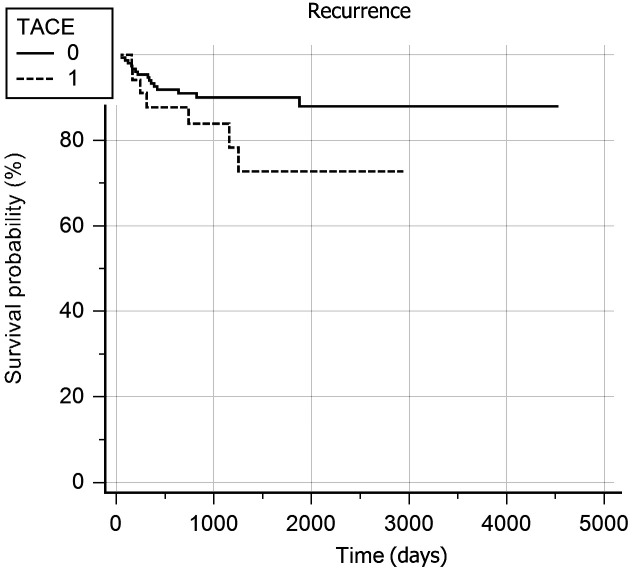
Kaplan-Meier analysis of hepatocellular carcinoma recurrence-free survival according to downstaging (transarterial-chemoembolization) procedure (0 – no, 1 – yes), *P* = 0.0677.

Even though the difference was not significant, one-year, three-year, and five-year tumor recurrence-free survival probabilities were more unfavorable in the downstaging group (90%, 81.6%, 76.5% vs 94%, 91.6%, 89.3%, *P* = 0.0677, Figure 5). The MC group had a longer time to HCC recurrence (median 1099.5 days, IQR 494-1962) compared with the downstaging group (median 807.5 days, IQR 262-1419, *P* = 0.0627), although the result was not significant.

### Factors associated with HCC-recurrence and recipient survival

The multivariate Cox regression analysis (backward stepwise method) included age (years), sex, TACE procedure, time from downstaging to MC and LT>3 months, number of TACE procedures, NT on imaging method before LT and on the explant (NT-3), MTD on imaging method before LT and on the explant (MTD-3), MC fulfillment on the explant (Milan–3), AFP level before LT, LT waiting time, MiVI, MaVI, and HCC recurrence (for survivors only).

**Figure 6:**
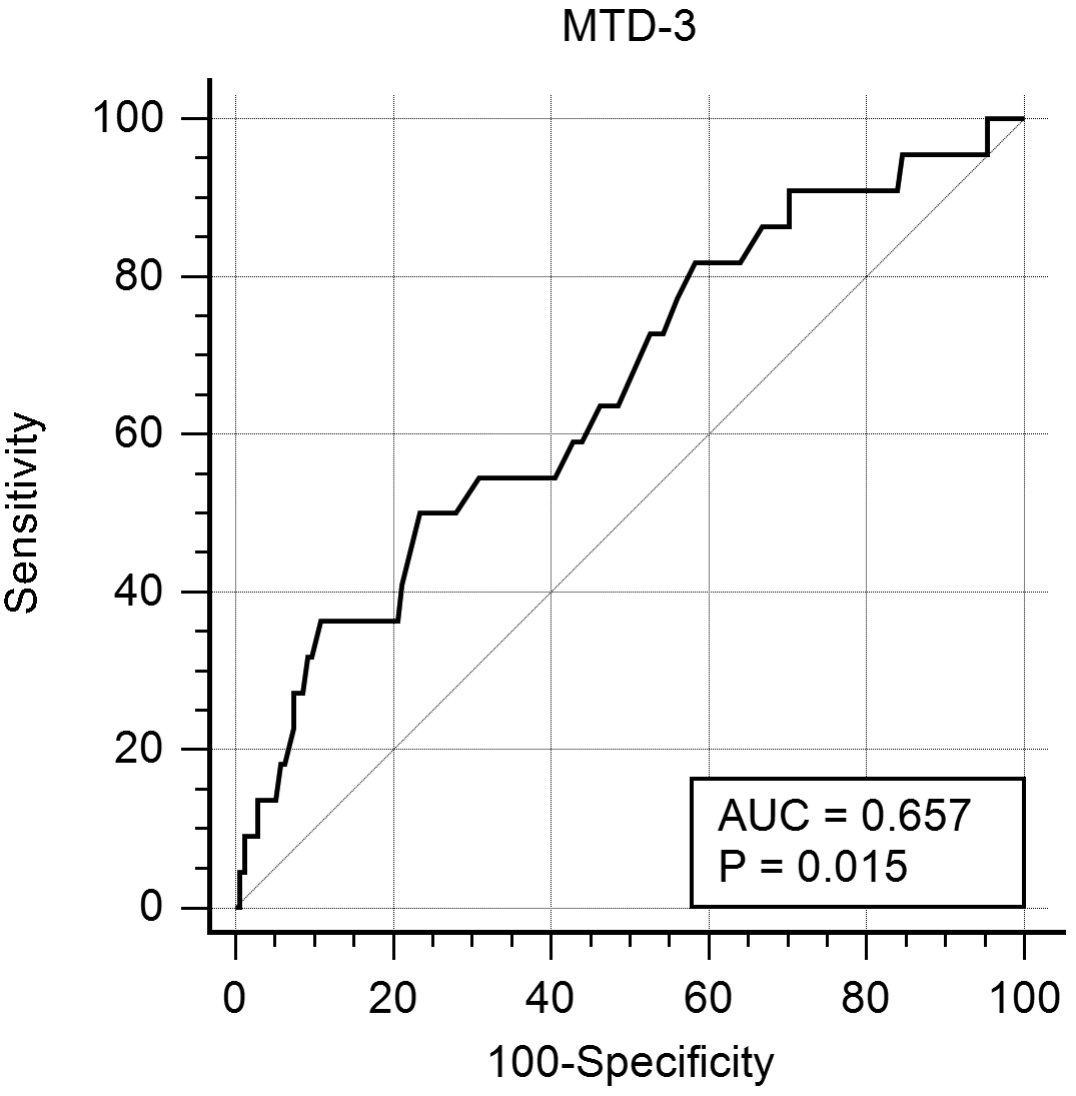
Receiver operating characteristic curve analysis indicating the optimal cut-off level of the sum of maximal viable tumor diameters on the explant (MTD-3) in the prediction of tumor recurrence to be >69 mm (sensitivity 50% and specificity 76.7%, *P* < 0.015, area under the curve [AUC] 0.657).

Only MTD-3 was a significant independent risk factor for tumor recurrence (HR 1.02; 95% CI 1.004-1.04; *P* = 0.01). ROC curve analysis indicated the optimal cut-off level for the sum of MTD-3 on the explant in the prediction of tumor recurrence to be >69 mm (sensitivity 50% and specificity 76.7%, area under the curve [AUC] 0.657, *P* < 0.015, Figure 6). The predictors of death were age, NT-3, and tumor recurrence (Table 2). The strongest predictor was HCC recurrence (HR 3.62; 95% CI 1.67-7.88; *P* = 0.001).

**Table 2:** Multivariate analysis of factors associated with recipient death outcome (Cox regression analysis)

Parameter	p	Hazard ratio	95% confidence interval
Age (years)	0.02	1.07	1.01 – 1.1
Number of tumors on explant finding (NT-3)	0.03	1.09	1.01-1.17
Hepatocellular carcinoma recurrence	0.001	3.62	1.67-7.88

### Comparison of the findings of pre-transplant HCC imaging and explant finding results

Overall, 36.9% of patients with HCCs did not fulfill the MC on explant findings. In 72.6% of patients both the tumor number and the sum of MTD were significantly higher, in 19.2% only the sum of MTD was significantly higher, and in 8.2% only the tumor number was significantly higher than on the imaging findings. The difference was considered significant if there was any new tumor nodule and/or a sum of MTD difference >10 mm. More patients in the downstaging group had discrepancies (52.6%) compared with those in the MC group (33.1%, *P* = 0.0254). In the 67.9% of MC patients, both the tumor number and the sum of MTD were significantly higher, in 20.8% only the sum of MTD was higher, while in 11.3% only tumor number was higher. In the downstaging group, the respective numbers were 85%, 15%, and 0%. Differences between groups in the type of discrepancies were not significant (*P* = 0.0665).

## Discussion

In this study, recipient survival and HCC recurrence-free survival did not differ between the groups, even though the downstaging group showed a trend toward more HCC recurrences and shorter time to HCC recurrence. The most important finding was that long-term recipient and HCC recurrence-free survival rate in the downstaging group were satisfactory, comparable with those in the MC group and previously published data on LT recipients fulfilling the MC (2). Most deaths (60%) occurred in the first year after LT, whereas only 32.4% were related to HCC recurrence. Since laboratory MELD, age, and rate of downstaged patients were comparable between the surviving and non-surviving recipients, the most likely explanation for the death outcomes unrelated to HCC is the recurrence of HCV infection. HCV patients were not treated with direct-acting agents before 2016, and many patients died or were re-transplanted due to HCV recurrence. The death rate of HCV-positive recipients was 28.6%, the highest when compared with other diseases.

The International Consensus Conference on LT for HCC recommended the evaluation criteria for downstaging procedure outcome based on the size and number of viable tumors. Even though macrovascular invasion and extrahepatic tumor spread are contraindications for downstaging procedure, there are still no well-defined criteria based on the upper limit of tumor nodules or diameters (6). Our center imposes no strict limits for the eligibility to downstaging procedure. In the downstaging group, 31.5% patients had more than 3 tumors (maximum 5), the median sum of MTD was 64 mm (range 10-141), and the greatest treated tumor diameter was 90 mm. The overall waiting time for LT was short (median 22.5 days) and comparable between the groups, as a consequence of high organ donation rate in Croatia. Only about 71.1% of recipients had downstaging-to-LT time on the waiting list longer than 3 months. Although we did not assess the overall effect of the TACE procedure on the eligibility for the LT, the majority of the studies on this issue are retrospective in design with discrepant results, without pre-defined tumor eligibility criteria for the procedure and a high drop-out rate (44%-76%) (7,13-15). Consequently, downstaging success rates are extremely variable (24%-90%), depending on the tumor burden, treatment modality, definition of response, liver disease severity, HCC progression rate, and availability of organs for LT (16-18). The majority of studies reported excellent first-year survival rates exceeding 90%, but variable five-year survival rates (70%-90%). Post-LT HCC recurrence-free survival rates at one and five years were 91% and 80%, respectively (18). Studies on downstaged patients, with strict inclusion criteria and mandatory waiting time before LT (proving better evaluation of disease response or stabilization and tumor biology) reported better LRT success, HCC recurrence rate, and survival, which were even equivalent to patients initially within the MC (7,19-21).

Even though data about risk factors affecting survival and HCC recurrence after downstaging are still emerging, our findings agree with the published data and are related to well-known factors associated with unfavorable outcomes after LT (2,9,19,22). The number of tumors on the explant, age, and HCC recurrence were significantly associated with death. The strongest predictor was HCC recurrence, amplifying the importance of pre-LT stratification of patients with the highest risk of disease recurrence. The sum of MDT on the explant was significantly related to HCC recurrence, whereas cut-off value of >69 mm diameter of viable tumor allowed optimal prediction of tumor recurrence on ROC analysis. Previous studies also highlighted a positive association of pre-LT tumor necrosis extent accomplished by LRT to a lesser HCC recurrence and a better survival (23-26).

In our study, no baseline tumor characteristic reliably predicted recipient survival and HCC recurrence-free survival. Beneficial tumor response to TACE, targeting the MC as a criterion for LT in our study, may be used in selection of patients with favorable tumor biological characteristics. Independent of tumor measurements, tumor response to downstaging is believed to ensure enough time for physicians to appraise its biological behavior and identify the patients at lowest risk of tumor progression and unfavorable post-LT outcomes (21,27,28). This is expected since favorable tumor response to LRT is often related to indicators of advantageous outcomes (ie, absence of MiVI and satellites, low tumor grading). Unfortunately, without tumor biopsy, these indicators are not available before LT. In our study, the rate of explant finding MiVI was comparable between the groups, which also supports the role of downstaging in the selection of patients with more auspicious tumor biological behavior. Since AFP level is related to a higher tumor burden and MiVI rate, the trend and final level of AFP at the end of downstaging procedure further elucidates tumor biology, although there is no consensus concerning the optimal AFP threshold before LT (4,19,29,30). In our center, there were no predefined criteria concerning the upper AFP cut-off at the time of listing to LT. However, 21.1% of patients in the downstaged group and 9.38% in the MC group had AFP level higher than 400 μg/mL. This suggests a higher rate of patients with unfavorable biology in the downstaged group, and may explain the trend toward more HCC recurrences in these patients.

Since both post-LT survival and HCC recurrence were best predicted with the explant finding of tumor number and size, we compared them with the pre-LT imaging assessment. Overall, 36.9% of explant findings did not fulfill the MC, significantly more in the downstaging group than in the MC group. Most patients had a discrepancy in both tumor number and the sum of MTD, followed by a discrepancy in the sum of MDT only. In the population with liver cirrhosis, both radiological methods have the sensitivity of <87% and the satisfying specificity of 78%-96% (4). Previous research also revealed a discrepancy in up to 25% of pretransplant radiological and explant pathology findings (31). As opposed to the tumor number, the size of HCC has a major prognostic role in most prediction models, with nodules <10 mm often not being considered in the analysis (32). Our criteria for discrepancy were very rigorous, which is a challenging approach having in mind the nodularity of cirrhosis.

Many medical teams use DEB-TACE for downstaging before LT. Compared with other LRTs, it is a well-standardized procedure and the beads likely lead to irreversible ischemia and reduced levels of vascular-endothelial-growth factor, which are negatively associated with tumor growth, metastasis formation, and poor survival (33). Due to the retrospective study design and the fact that all downstaged patients were treated with TACE, we were unable to compare different LRT procedures and treatment selection criteria. We were also unable to evaluate the intention-to-treat downstaging procedure outcomes, and consequent LT rate, to be able to investigate the effects of mandatory waiting time before LT and the factors predicting the waiting list dropout. Studies with very strict inclusion criteria and LRT protocol are needed to better define an optimal downstaging procedure and pre-LT factors related to a favorable outcome.

The results of our and previous studies show that even patients initially exceeding the MC when successfully downstaged can attain post-LT recipient and HCC recurrence-free survival comparable to patients initially meeting the MC. This might be related to the positive effects of downstaging when it comes to selection of the tumors with most favorable biological behavior. Even though the two patient groups did not significantly differ in survival, our results also revealed that non-selective criteria for downstaging can result in a trend toward higher tumor recurrence rates after LT. This implies that, except limitations in the reliability of imaging methods, there are other unknown pre-LT factors related to unfavorable outcomes of downstaged patients. In further studies, conventional criteria for defining the success of downstaging before and outcome after LT are likely to be replaced with composite criteria that combine multiple surrogates of tumor biology. Until such criteria are available, in order to achieve maximum success of downstaging procedure accompanied with favorable LT outcomes, the procedure should be performed exclusively within strictly defined protocols.
